# Risk-Based Evaluation of Total Petroleum Hydrocarbons in Vapor Intrusion Studies

**DOI:** 10.3390/ijerph10062441

**Published:** 2013-06-13

**Authors:** Roger Brewer, Josh Nagashima, Michael Kelley, Marvin Heskett, Mark Rigby

**Affiliations:** 1 Hawaii Department of Health, 919 Ala Moana Blvd Room 206, Honolulu, HI 96814, USA; E-Mail: josh.nagashima@doh.hawaii.gov; 2 EA Engineering, Science and Technology, 615 Piikoi St #515, Honolulu, HI 96814, USA; E-Mail: mkelley@eaest.com; 3 Geotek Hawaii, Inc., P.O. Box 1555, Pearl City, HI 96782, USA; E-Mail: mhesketts@mac.com; 4 Parsons Corporation, 10235 South Jordan Gateway, Suite 300, South Jordan, UT 8409, USA; E-Mail: mark.rigby@parsons.com

**Keywords:** petroleum, TPH, carbon ranges, benzene, soil gas, soil vapor, vapor intrusion, risk assessment

## Abstract

This paper presents a quantitative method for the risk-based evaluation of Total Petroleum Hydrocarbons (TPH) in vapor intrusion investigations. Vapors from petroleum fuels are characterized by a complex mixture of aliphatic and, to a lesser extent, aromatic compounds. These compounds can be measured and described in terms of TPH carbon ranges. Toxicity factors published by USEPA and other parties allow development of risk-based, air and soil vapor screening levels for each carbon range in the same manner as done for individual compounds such as benzene. The relative, carbon range makeup of petroleum vapors can be used to develop weighted, site-specific or generic screening levels for TPH. At some critical ratio of TPH to a targeted, individual compound, the overwhelming proportion of TPH will drive vapor intrusion risk over the individual compound. This is particularly true for vapors associated with diesel and other middle distillate fuels, but can also be the case for low-benzene gasolines or even for high-benzene gasolines if an adequately conservative, target risk is not applied to individually targeted chemicals. This necessitates a re-evaluation of the reliance on benzene and other individual compounds as a stand-alone tool to evaluate vapor intrusion risk associated with petroleum.

## 1. Introduction

Much emphasis has been placed in the past ten-plus years on the potential intrusion of chlorinated solvent vapors into buildings from underlying contaminated soil and groundwater. The study of vapor intrusion associated with subsurface releases of petroleum fuels is, in comparison, still in its infancy. The complex chemistry of petroleum fuels and the difficulty of predicting the fate and transport of vapors in the subsurface hamper the development of easy-to-use guidance that can be applied under multiple site scenarios. This paper addresses the first issue. Other efforts are currently underway to compile field data and address the second topic. 

Petroleum-contaminated soil and groundwater are traditionally assessed in terms of Total Petroleum Hydrocarbons (TPH) and targeted, individual compounds such as benzene, toluene, ethylbenzene, xylenes and naphthalene (BTEXN). The buildup of methane vapors at petroleum-release sites can also pose potential fire and explosion hazards. This topic is beyond the scope of this paper, however. As noted in Table 1, non-specific, aliphatic and aromatic compounds collectively quantified as TPH make up the overwhelming mass of liquid fuels. Risk-based assessment of TPH in soil is well established and in use in numerous states [1,2,3,4,5,6,7,8,9]. While relatively straight forward, the quantitative inclusion of TPH in vapor intrusion investigations is less-well established and few papers and guidance documents have been published on this topic [10,11]. Some states require an assessment of potential vapor intrusion hazards associated with both TPH and individually targeted compounds at sites where long-term, *in situ* management of petroleum-contaminated soil or groundwater is proposed [12]. 

**Table 1:** Range of current and past BTEX and naphthalene (BTEXN) concentrations in petroleum fuels.

Chemical	Gasolines ^1^	Diesel ^2^	Residuel Fuels ^3^
Benzene	0.1–4.9%	0.003–0.1%	0.06–0.1%
Ethylbenzene	0.1–3%	0.007–0.2%	
Toluene	1–25%	0.007–0.7%	0.1–0.2%
Xylenes	1–15%	0.02–0.5%	0.2–0.3%
Naphthalene	<1%	0.01–0.8%	

^1^ Gasoline ranges after [1,13,14]; ^2^ Diesel #2 [1]; ^3^ Lubricating and motor oil [1].

This paper considers a series of key questions related to potential vapor intrusion concerns posed by TPH in contaminated soil and groundwater: (1) “How are the chemistry and toxicity of petroleum vapors characterized and evaluated?”; (2) “What is the composition of vapors emitted from fresh fuels and petroleum-contaminated soil and groundwater in terms of TPH and traditionally targeted, individual compounds such as BTEXN?”; (3) “What is the chemical makeup of the TPH component of these vapors in terms of aliphatic and non-BTEXN aromatic carbon ranges?”; (4) “What is the toxicity of the TPH in terms of the weighted, carbon range composition?”; (5) At what critical ratio of TPH to an individual compound will the former begin to drive relative vapor intrusion risk over the latter, due to its overwhelming dominance of soil vapors?”; (6) “Under what site scenarios might vapor intrusion be driven by TPH rather than a individual compound such as benzene?”

The methodology described in this paper consists of six components: (1) Categorization of petroleum fuels into broad types based on the number of carbon atoms in compounds that typify the fuels, (2) Characterization of the non-BTEXN, TPH component of the fuels in terms of aliphatic and aromatic “carbon ranges”, (3) Assignment of inhalation toxicity factors to volatile carbon ranges, (4) Calculation of risk-based, carbon range screening levels for indoor air and soil vapor, (5) Calculation of weighted screening levels for TPH based on the carbon range makeup of petroleum vapors, and (6) Calculation of the “critical ratio” of TPH in soil vapor to an individual chemical (e.g., benzene), at which point TPH will drive vapor intrusion risk over the individual compound even when a conservative, target risk is applied to the latter. These tools are then applied to two example sets of soil vapor data, the first associated with releases of gasolines and the second from sites associated with releases of middle distillates. The results are used to evaluate the relative role of TPH in vapor intrusion in comparison to traditionally targeted compounds such as benzene. 

## 2. Methods

### 2.1. Categorization of Fuel Types

**Figure 1:**
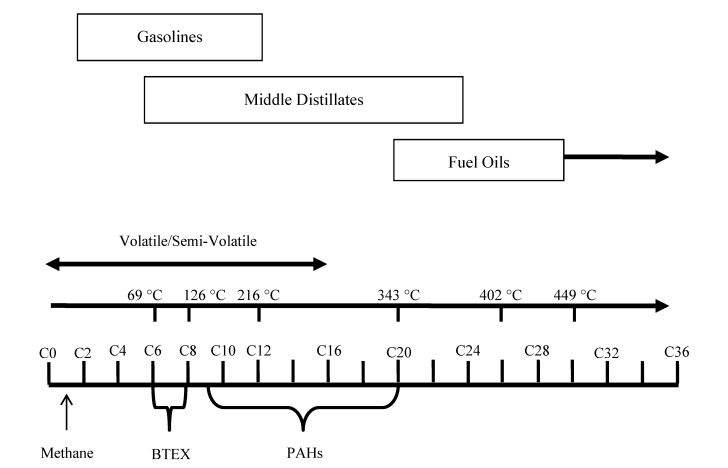
Composition of typical petroleum fuels with respect to the number of carbon molecules in individual compounds.

Petroleum fuels can be broadly categorized as “gasolines”, “middle distillates” and “residual fuels”, following the methodology used by the American Petroleum Institute [15]. The chemistry of these fuels has been extensively studied [1,16]. These categories in part reflect the number of carbon atoms in individual compounds that characterize the fuels (Figure 1). Compounds with less than approximately sixteen carbon atoms are considered to be “volatile” to “semi-volatile,” with a propensity to partition into the vapor phase under ambient conditions. These compounds, which include a host of short-chain, aliphatic chemicals collectively measured as “TPH” as well as aromatic chemicals such as benzene, toluene, ethylbenzene, xylenes and naphthalene, are the primary target of vapor intrusion investigations. A summary of the BTEXN composition of petroleum fuels is provided in Table 1. Non-specific, TPH aliphatic and aromatic compounds comprise the remainder of the fuels.

Gasolines, including automotive gasoline and older jet fuels such as AVGAS, are dominated by “lighter” compounds with six to twelve carbon atoms. This causes gasolines to be highly volatile in comparison to other types of fuels. The amount of benzene, toluene, ethylbenzene and xylenes in gasolines can vary dramatically, from just a few percent to greater than 20%, depending on the refiner, the desired performance of the fuel and the historical time period that the fuel was produced (see Table 1). The benzene content of automotive gasolines can in particular vary significantly, from less than 0.1% to greater than 5% [14]. Recent regulations in the United States limit the average amount of benzene in gasolines to less than one-percent after the year 2011 in order to reduce health effects from exposure to vapors and exhaust [17,18]. Older formulations of jet fuels and aviation gasoline likewise contained a relatively minor amount of benzene [13].

Middle distillate fuels (e.g., diesel, kerosene, JP-8 jet fuel, *etc.*) are dominated by hydrocarbon compounds with approximately nine to twenty-five carbon atoms and a relatively minor fraction of BTEX (see Table 1). Naphthalene, a suspected carcinogen, can comprise up to one-percent of these fuels. As a result, these fuels are less volatile than gasolines. Middle distillate fuels do, however, include a minor but important component of lighter and more volatile aliphatic compounds and, to a lesser extent, aromatic compounds. As discussed below, these aliphatic compounds not surprisingly dominate vapors emitted from these fuels under ambient conditions. Older jet fuels such as JP-4 are a mixture of gasoline and kerosene and again, while less volatile than gasolines, display a distinct vapor phase that is dominated by lighter-range aliphatic and aromatic compounds.

Residual fuels (e.g., Fuel Oil Nos. 4, 5, and 6, lubricating oils, “waste oils”, asphalts, *etc.*) are characterized by complex, polar PAHs and other high molecular weight hydrocarbon compounds with carbon ranges that generally fall between C24 and C40. Residual fuels lack a significant amount of volatile compounds (e.g., see Table 1) and, aside from the potential generation of methane, are generally assumed to pose a minimal vapor intrusion risk. This subsequent focus of this paper will therefore be on vapors associated with gasolines and middle distillate fuels.

### 2.2. Characterization Total Petroleum Hydrocarbons Using Carbon Ranges

Understanding the chemical makeup of the TPH component of petroleum fuels and more importantly the vapors emitted from these fuels is important, first step to evaluate the role of these compounds in vapor intrusion. Petroleum is a complex mixture of hundreds of different compounds composed of hydrogen and carbon or “hydrocarbons”. These compounds can be collectively grouped into “aromatic” and “aliphatic” carbon ranges, based in part on the number of carbon atoms in each compound [1].

Compounds formed by single or multiple, six-carbon rings are referred to as “aromatic”. Aromatic compounds include the familiar chemicals benzene, toluene, ethylbenzene and xylenes (BTEX) as well as naphthalene and other “polyaromatic” hydrocarbons. A small percentage of additional, aromatic compounds are included in the TPH component of fuels. These include alkylated compounds such as trimethylbenzene, which although sometimes reported by laboratories as part of an environmental investigation are not traditionally evaluated in human health and ecological risk assessments as individual chemicals.

Compounds formed by chains or non-aromatic rings of carbon and hydrogen are referred to as “aliphatic” and include such chemicals as pentane, hexane and octane. These compounds make up the bulk of petroleum fuels [1]. A host of additional terms are used to classify aliphatic compounds in more detail, depending for example on the presence or absence of ring structures, nature of carbon bonds, saturation with hydrogen and overall chemical structure (e.g., “alkanes”, “alkenes”, “olefins” and “cycloalkanes”, *etc.*). 

Evaluation of each individual, TPH-related aromatic and aliphatic compound as part of an environmental investigation is not feasible or practical due to the large number of compounds involved and the lack of physiochemical and toxicological information for these chemicals. The TPH component of petroleum is instead evaluated in terms of “carbon ranges” of aliphatic and aromatic compounds. Carbon ranges are defined by groups of aliphatic or aromatic compounds that exhibit similar physiochemical and, presumably, toxicological characteristics. Carbon range fractions designated by Massachusetts are the most commonly referenced in the United States (see Figure 1) [19]:

- C5-C8 aliphatics;
- C9-C12 aliphatics;
- C13-C18 aliphatics;
- C19-C36 aliphatics;
- C9-C10 aromatics;
- C11-C22 aromatics.

These carbon range groups represent a consolidation and simplification of a larger number of ranges originally published by the TPH Criteria Working Group, an environmental consortium of regulators, consultants and oil company experts convened to develop a more comprehensive, risk-based approach for the evaluation of petroleum-contaminated soil and groundwater [20]. This was done in part on available toxicity factors for individual ranges. Compounds that fall within the C5-C8 aliphatic carbon range are the most volatile, although C9-C12 aliphatics and C9-C10 aromatics also fall in this category. Compounds that fall within the C13-C18 aliphatic and C11-C22 aromatic carbon ranges are considered to be “semi-volatile.” Aliphatic compounds with greater than 18 carbon atoms and aromatic compounds with greater than ten carbon atoms are not considered to be volatile. Carbon ranges can also be defined in terms of “Equivalent Carbons,” based on the boiling point of individual compounds [5,20].

As discussed below, assignment of physiochemical and toxicological parameter values to individual carbon ranges allows for quantitative inclusion of TPH in environmental risk assessments in the same manner as individual compounds. This includes the development of risk-based screening levels for water, soil, soil vapor and indoor air. This approach was first developed by the Total Petroleum Hydrocarbon Criteria Working Group [20]. Guidance on the use of carbon-range approaches to quantitatively evaluate the non-BTEX, TPH component of petroleum-contaminated media was subsequently developed by a number of state agencies (e.g., [2,3,4,5,6,8,9]).

The bulk chemistry of petroleum fuels in terms of TPH carbon ranges and commonly targeted, individual, aromatic compounds is summarized in Table 2 (after [2,21]). Aliphatic compounds dominate both the TPH and overall component of petroleum fuels. Gasolines are dominated by C5-C8 aliphatics and C9-C12 aromatics, although the proportion of the latter can vary widely depending on the fuel blend. Residual fuels are dominated by longer-chain aliphatics and a lesser amount of polyaromatic hydrocarbons.

**Table 2:** Example carbon range makeup of non-BTEXN, TPH component of petroleum fuels (exact carbon range makeup of individual fuels will vary).

Carbon Range	Gasolines ^1^	Diesel ^1^	Residual Fuels ^2^
C5-C8 aliphatics	45%	<1%	<1%
C9-C18 aliphatics	12%	35%	<1%
C19+ aliphatics	<1%	43%	75%
C9-C12+ aromatics	43%	22%	25%

^1^ Indiana Department of Environmental Management [21]; ^2^ Massachusetts Department of Environmental Protection [2].

Physiochemical constant values published by Massachusetts [2], currently most in use in the US, are summarized in Table 3. Values for BTEX and naphthalene are included for comparison [22]. The chemical makeup of vapors emitted from petroleum fuels is predictable based on the composition of the fuels and the theoretical partitioning of chemicals into sorbed, dissolved and vapor phases upon release to the environment [23]. Vapors emitted from fresh gasolines can be predicted to be dominated by C5-C8 aliphatics (and C2-C4 aliphatics, if present) based both on the abundance and relative volatility of these compounds, with a variable but lesser amount of BTEX and other aromatic compounds depending on the specific fuel blend (see also [24] and [25]). While less volatile than gasolines, diesel and other middle distillate fuels contain variable amounts of C5-C8 aliphatics and a relatively large component of C9-C18 aliphatics (see Table 2). These compounds should again dominate vapors emitted from the fuels. The relative proportion of C5-C8 to C9-C12 aliphatics in vapors will depend in part on the original composition of the fuel (see also [26]). The fraction of BTEX in the vapors should be significantly smaller than for gasolines, given their lower relative abundance.

This general makeup of petroleum vapors is indeed observed in the case studies presented later in this paper. As discussed in the case studies, soil vapor samples from some of the middle distillate-release sites contain a significant proportion of C5-C8 “gasoline-range” compounds. Requesting a lab to test a sample for “diesel-range” hydrocarbons as the sum of C9 and higher compounds is reasonable for soil, since this fraction dominates the liquid fuel and should similarly dominate the TPH present in the soil. Requesting that TPH be quantified in terms of traditional, diesel-range compounds for soil vapor could result in a significant underreporting of the total TPH present, however. Laboratories should instead be requested to report TPH in soil vapors simply as the sum of C5 to C12 hydrocarbons for both gasoline- and middle distillate-contaminated sites. Testing for additional, heavier vapor-phase compounds (e.g., C13+ aliphatics) may also be necessary. This is discussed further in the following section, as well as in the example case studies.

**Table 3:** Default physiochemical constants for BTEXN and TPH carbon ranges.

Chemical/Carbon Range ^1^	Molecular Weight	Vapor Pressure (atms)	Solubility in Water (mg/Lf)	Henry’s Constant (unitless)	Partition Coeff, k_oc_ (cm^3^/g)	Diffusion Coefficient (cm^2^/s)	
						air	water
Benzene	78	0.1	1,790	0.23	146	0.09	1 × 10^−^^5^
Ethylbenzene	106	0.01	169	0.32	446	0.068	8.5 × 10^−^^6^
Toluene	92	0.04	526	0.27	234	0.078	9.2 × 10^−^^6^
Xylenes	106	0.01	161	0.29	375	0.068	8.4 × 10^−^^6^
Naphthalene	128	1.0 × 10^−^^4^	30	0.018	1,540	0.06	8.4 × 10^−^^6^
C5-C8 Aliphatics	93	0.1	11	54	2,265	0.08	1 × 10^−^^5^
C9-C12 Aliphatics	149	8.7 × 10^−^^4^	0.07	65	150,000	0.07	1 × 10^−^^5^
C13-C18 Aliphatics	170	1.4 × 10^−^^4^	3.5 × 10^−^^4^	69	680,000	0.07	5.0 × 10^−^^6^
C19-C36 Aliphatics	280	1.1 × 10^−^^6^	1.5 × 10^−^^6^	110	4.0 × 10^−^^8^		
C9-C10 Aromatics	120	2.9 × 10^−^^3^	51	0.33	1,778	0.07	1 × 10^−^^5^
C11-C22 Aromatics	150	3.2 × 10^−^^5^	5.8	0.03	5,000	0.06	1 × 10^−^^5^

^1^ Constants for BTEXN from USEPA RSL guidance [22]; vapor pressures from TOXNET [27]; Carbon range values from Massachusetts DEP [2] except C13-C18 Aliphatics (based on EC > 12–16) and C19-C36 Aliphatics (based on EC > 16–35 aliphatics) [20].

### 2.3. Assignment of Inhalation Toxicity Factors to Carbon Ranges

Key to the risk-based assessment of TPH in vapor intrusion investigations is the assignment of inhalation toxicity factors or “Reference Concentrations (RfC)” to individual, volatile carbon ranges. A summary of published inhalation toxicity factors for carbon ranges is presented in Table 4. Lower RfCs reflect progressively increasing toxicity (*i.e.*, less of the chemical is required to result in a health effect).

The TPH Criteria Working Group published an extensive overview of the carbon range chemistry of petroleum fuels in the late 1990s and assigned preliminary toxicity factors to each fraction [28]. The US Department of Health and Human Services quickly published updated guidance in 1999 [29]. The Massachusetts Department of Environmental Protection published initial guidance during the same time period and last updated their factors for carbon range fractions in 2003 [19]. The Washington Department of Ecology published toxicity factors for TPH carbon ranges in 2005 and 2006 [5]. In 2009, the California EPA Department of Toxics Substances Control published guidance and proposed toxicity factors similar to those proposed by MADEP [30]. The USEPA National Center for Environmental Assessment published a detailed review of TPH carbon range toxicity and recommended Provisional Peer-Reviewed Toxicity Values (PPRTVs) in 2009 [16].

**Table 4:** Published inhalation toxicity factors for petroleum aliphatic and aromatic carbon ranges (listed in order of publication).

Reference	RfC (mg/m^3^)	RfC (µg/m^3^)
TPH Criteria Working Group [28]		
(C5-C8) Aliphatics	18.4	18,400
(C9-C18) Aliphatics	1.0	1,000
(C9-C16) Aromatics	0.2	200
USDHHS ^1^ [29]		
(C5-C8) Aliphatics	2.2	2,200
(C9-C18) Aliphatics	0.3	300
(C9-C16) Aromatics	0.01	10
Massachusetts DEP [19]		
(C5-C8) Aliphatics	0.2	200
(C9-C18) Aliphatics	0.2	200
(C9-C18) Aromatics	0.05	50
Washington DOE ^2^ [5]		
(C5-C8) Aliphatics	6.0	5,950
(C9-C16) Aliphatics	0.3	298
(C9-C10) Aromatics	0.399	399
(C11-C12) Aromatics (naphthalene)	0.003	3.0
(C13-C16) Aromatics	0.2	175
CalEPA-DTSC ^3^ [30]		
(C5-C8) Aliphatics	0.7	700
(C9-C18) Aliphatics	0.3	300
(C9-16) Aromatics	0.05	50
USEPA^4^ [16]		
(C5-C8) Aliphatics (noncancer)	0.6	600
(C9-C18) Aliphatics	0.1	100
(C9-C16) Aromatics	0.1	100

^1^ ATSDR C5-C8 aliphatics RfC converted to 2.2 mg/m^3^ from 0.6 ppm based on hexane molecular weight of 86; C9-C16 aromatics RfC converted to 0.01 mg/m^3^ from 0.002 ppm based on naphthalene molecular weight of 128; ^2^ Washington DOE Inhalation Reference Dose extrapolated to a Reference Concentration: using RfC (mg/m^3^) = RfD (mg/kg-day) × 70 kg × (1/20m^3^-day); ^3^ California EPA toxicity factors withdrawn in 2010 pending review of additional data; ^4^ USEPA toxicity factors selected for calculation of risk-based indoor air and soil vapor screening levels.

The variability of published toxicity factors for individual carbon ranges is important, since this directly affects the estimated risk (or more appropriately noncancer hazard) posed by TPH in a vapor intrusion study. Of particular interest is the RfC assigned to C5-C8 aliphatics, since as discussed above and noted in case studies below, these compounds tend to dominate the TPH component of petroleum vapors. For example, the inhalation RfC published by USEPA (600 μg/m^3^) is less conservative (*i.e.*, higher) than the correlative toxicity factor published by Massachusetts (200 μg/m^3^) but an order of magnitude or more lower than toxicity factors published by the State of Washington (equal to 5,950 μg/m^3^) and the earlier toxicity factor the TPH Criteria Working Group (18,400 μg/m^3^).

Based on a review of published guidance, the State of Hawaii [8] opted to incorporate PPRTVs for volatile carbon ranges published by the USEPA [16]. Conclusions drawn from the case studies presented would necessarily differ based on the toxicity factors selected for the carbon ranges. Full consensus is rarely if ever reached on toxicity values for specific chemicals, however, including toxicity factors posted to USEPA’s IRIS database—considered to be the most supportable and defensible database available. States as well as USEPA routinely draw on available information for assessment of the health risk posed by chemicals that are not currently listed in IRIS. Indeed, Regional Screening Levels published in USEPA’s guidance document are based in part or entirely on PPRTV toxicity factors for over one-hundred of the chemicals listed [22].

A summary of the PPRTV inhalation toxicity factors [16] for carbon ranges and inhalation toxicity factors for BTEXN is provided in Table 5. The toxicity factors address systemic, noncancer health hazards. Cancer risk is assumed to be driven by well-studied, individual compounds such as benzene, ethylbenzene and naphthalene [8,22].

**Table 5:** Inhalation toxicity factors for targeted VOCs and carbon range fractions.

Chemical	IUR ^1^ (µg/m^3^)^−^^1^	RfC ^2^ (µg/m^3^)
Benzene	7.8E−06	30
Ethylbenzene	2.5E−06	1,000
Toluene		5,000
Xylenes		100
Naphthalene	3.4E−05	3.0
C5-C8 aliphatics		600
C9-C18 aliphatics		100
C9+ aromatics		100

^1^ Inhalation Unit Risk [22]; ^2^ Reference Concentration; BTEXN RfCs from USEPA [22]; Carbon Range RfCs from USEPA [16].

### 2.4. Calculation of Risk-Based Air and Soil Vapor TPH Screening Levels

Calculation of risk-based screening levels for TPH in indoor air and soil vapor or direct inclusion in human-health risk assessments is relatively straight forward following assignment of inhalation toxicity factors to volatile carbon ranges. Accurate quantitative evaluation of vapor intrusion risks based on soil and groundwater data is much more difficult, as discussed earlier, due to the variability of biodegradation and attenuation processes on a site-by-site basis. This likewise impedes the development of meaningful TPH screening levels for other than subslab or very shallow soil vapors [8]. The collection of sub-slab soil vapor samples helps to minimize uncertainty regarding the fate and transport of petroleum vapors in the subsurface, since these vapors can be assumed to undergo minimal, additional attenuation prior to intruding into an overlying building.

For the purposes of this paper, the PPRTV toxicity factors published by the USEPA in 2009 [16] were selected for calculation of example, indoor air and subslab soil vapor screening levels for individual carbon ranges (see Table 4). The development of indoor air and subslab, soil vapor screening levels for vapor intrusion can be condensed into three relatively simple steps: (1) Calculation of a target indoor-air goal based on the assigned toxicity factor and default exposure assumptions (e.g., exposure frequency and duration); (2) Assignment of an indoor air: subslab soil vapor attenuation factor based on a comparison of vapor flow rates into a building and air flow rates through the building and (3) Calculation of a soil vapor screening level. A summary of these steps is provided below.

Indoor air screening levels can be calculated using the ambient air equations presented in the USEPA Regional Screening Level guidance [22]:

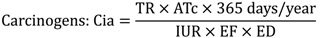
(1)

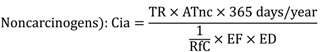
(2)
where:

Cia = Indoor air concentration (µg/m^3^);
TR = Cancer Target risk (10^−^^6^, unitless);
THQ = Noncancer Target Hazard Quotient (1.0, unitless);
ATc = Carcinogen Averaging Time (70 years);
ATnc = Noncancer Averaging time (30 years);
IUR = Cancer Inhalation Unit Risk (chemical-specific, (µg/m^3^)^−^^1^)
RfC = Noncancer Reference Concentration (chemical-specific, µg/m^3^);
EF = Exposure frequency (350 days/year); and
ED = Exposure duration (30 years).

Default exposure and target risk parameter values used for calculation of the indoor air screening levels are noted above and based on residential exposure assumptions used for development of the USEPA RSLs [22].

Example indoor-air screening levels for BTEX, naphthalene and carbon ranges based on the above equations and exposure assumptions and toxicity factors noted in Table 4 are presented in Table 6. Noncancer screening levels for benzene, ethylbenzene and naphthalene are not shown, since they would be higher than and over ridden by cancer-based screening levels. A target excess cancer risk was of 10^−^^6^ was used for carcinogenic VOCs. A target Hazard Quotient of 1.0 was used for noncancer-based screening levels. Note that these screening levels do not directly take into account cumulative risk posed by the potential presence of other chemicals with similar health effects. This is less of an issue for screening levels based on cancer risk, since they are set at the most conservative end of the target risk range of 10^−^^4^ to 10^−^^6^. Consideration of potential cumulative risk is especially important for screening levels based on noncancer concerns, however, since no safety margin is included (*i.e*., maximum target Hazard Index often set at 1.0) [22]. 

Calculation of a subslab soil vapor-to-indoor air attenuation factor (AF) essentially reduces to:
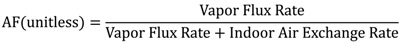
(3)

For the purposes of this paper, indoor air-soil vapor attenuation factors of 0.001 (residential scenario) and 0.0005 (commercial/industrial scenario) published by the state of Hawaii were referred to for calculation of soil vapor screening levels [8]. These attenuation factors are based on building ventilation rates typical of tropical and Mediterranean climates and may not be appropriate for use in colder regions where buildings are heated for much of the year, but are adequate for demonstration purposes. The rapid breakdown of aliphatic compounds under aerobic conditions is anticipated to significantly lower the persistence of aliphatic compounds in indoor air in comparison to chlorinated solvents and play an important role in the reduction of long-term, vapor intrusion risk [31]. A detailed discussion of this issue is beyond the scope of this paper, however, and the noted attenuation factors are presented for use as examples only.

**Table 6:** Example indoor air and subslab, soil vapor screening levels for petroleum-related chemicals.

Chemical	Indoor Air ^1^		Subslab Soil Vapor ^2^	
	Residential (µg/m^3^)	Commercial/Industrial (µg/m^3^)	Residential (µg/m^3^)	Commercial/Industrial (µg/m^3^)
Benzene	0.31	1.6	310	3,200
Ethylbenzene	0.97	4.9	970	9,800
Toluene	5,200	22,000	5,200,000	44,000,000
Xylenes	100	440	100,000	880,000
Naphthalene	0.072	0.36	72	720
C5-C8 aliphatics	630	880	630,000	1,760,000
C9-C18 aliphatics	100	150	100,000	300,000
C9-C16 aromatics	100	150	100,000	300,000

^1 ^Based on target cancer risk of 10^−^^6^ (benzene, ethylbenzene, naphthalene) or noncancer Hazard Quotient of 1.0 (toluene, xylenes and carbon range compounds); ^2 ^Based on indoor air-soil vapor (subslab) attenuation factors of 0.001 for residential structures and 0.0005 for commercial/industrial structures (after [8]; for example only).

Soil-gas screening levels (C_sg_) are subsequently calculated as:

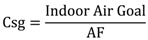
(4)

Example subslab soil-gas screening levels for BTEXN and volatile aliphatic and aromatic carbon ranges, and TPH using the above approach are included in Table 6.

Screening levels for C5-C8 aliphatics are the least stringent of the carbon range compounds (e.g., indoor air screening level 630 µg/m^3^), reflecting the higher inhalation Reference Concentration assigned to this fraction of 600 µg/m^3^. Screening levels for C9-C18 aliphatics and C9-C16 aromatics are most stringent, reflecting the lower Reference Concentration of 100 µg/m^3^ common to both fractions and generating an identical indoor air screening level of 100 µg/m^3^, after rounding. The screening levels are based on a target, noncancer hazard quotient of 1.0.

The example soil-gas screening levels do not take into account an expected decrease in vapor concentrations over time due to biodegradation and source area depletion and can be overly conservative for sites with limited contamination. Mass-balance approaches can be used to estimate maximum, average vapor concentrations over the assumed exposure duration based on an estimate of the mass of the chemical present in the source area.

As discussed later in this paper, a comparison of TPH carbon range screening levels to screening levels for individual compounds provides a useful tool to determine if the former might drive vapor intrusion risk over the latter at a site. Calculation and use of a single, TPH screening level weighted with respect to the representative (or assumed), carbon range makeup of petroleum vapors at a site will significantly speed up this process, however, and avoid the need to collect expensive carbon range data for every sample. Variability in TPH composition within a site due to biodegradation and other factors that affect partitioning (e.g., soil moisture and organic carbon content) can complicate this assessment, however. In these cases use of the most conservative, weighted RfC calculated for the site may be warranted.

### 2.5. Calculation of Weighted, TPH Screening Levels

The use of TPH soil vapor data is generally preferable for initial screening of petroleum-contaminated sites due to the added cost and the currently limited number of laboratories that can provide vapor-phase carbon range data. The following equation can be used to calculate weighted inhalation Reference Concentration (RfC) for TPH based on the site-specific carbon range makeup of TPH in soil vapor or indoor air [8,10]:

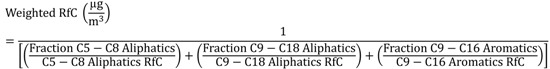
(5)

This approach can be used to calculate weighted TPH toxicity factors (RfCs) and associated indoor air and soil vapor screening levels based on either site-specific data or an assumed, carbon range makeup of TPH vapors for a specified fuel type.

Very few studies have been published regarding the detailed, carbon range makeup of vapors from common petroleum fuels. Carbon range data presented in the USEPA Petroleum Vapor Intrusion (PVI) database were used to approximate the chemistry and ultimately the weighted toxicity of TPH vapors associated with gasolines (see paper [Supplementary-material ijerph-10-02441-s001]Supplementary Material) [32]. The database is intentionally biased toward gasoline-contaminated sites, although as noted later in this paper significantly high TPH:Benzene ratios for some samples suggest that data from diesel-contaminated sites may also be included.

For illustration purposes in this paper, the average carbon range makeup of the data presented in the USEPA database was used to approximate the carbon range makeup of gasoline vapors in general. The review was limited to samples from gasoline-only sites with paired TPH and benzene data and reported concentrations of TPH >1,000 µg/m^3^. The latter filter was included in order to limit potential biases due to laboratory detection limits or interference from background, petroleum vapors associated with unrelated, indoor or outdoor sources [2]. Apparent duplicate sample data for some sites was also ignored (*i.e.*, identical concentrations of TPH and benzene). A total of 364 samples from 48 sites met these criteria (see paper supplement). Carbon range data were included for 35 samples from ten of the original 48 sites. The average carbon range composition of TPH in the samples is 77.3% C5-C8 aliphatics, 15.4% C9-C12 aliphatics and 7.3% C9-C10 aromatics. The aliphatic and aromatic makeup of the samples spans a broad range, with the median composition more biased toward C5-C8 aliphatics than the mean composition. The proportion of C5-C8 aliphatics in the samples ranges from 12% to 100%, with a median of 88%. The proportion of C9-C12 aliphatics ranges from 0% to 77%, with a median of 10%. The proportion of C9-10 aromatics ranges from 0% to 55%, with a median of median 2%.

For the purposes of this example, the average carbon range makeup of the samples in the USEPA PVI database report [32] was used to generate a weighted, TPH RfC for gasoline vapors of 279 µg/m^3^ using Equation 5 above:

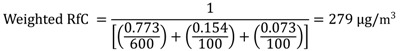
(6)

Risk-based screening levels and associated “critical ratios” for TPH vapors associated with gasoline (TPHg) based on this example RfC are used later in this paper to evaluate a soil vapor database for gasoline-contaminated sites published by the USEPA.

Even less data are available for the carbon range makeup of vapors from diesel and other middle distillates. A limited, field study by the Hawaii Department of Health (HDOH) identified a highly variable composition of vapors for diesel fuels and jet fuels, with C5-C8 aliphatics dominating at some sites and C9-C12 aliphatics dominating at others [10]. Data from this study are discussed later in this paper. The study intentionally focused on diesel- and middle distillate-contaminated sites, as a compliment to the developing, USEPA database for gasoline-contaminated sites. Sorbent tube data suggested an insignificant amount of C13-C18 aliphatics and C11-C16 aromatics in the samples. For the purposes of this paper, the hypothetical TPH composition for diesel and other middle distillate vapors of 25% C5-C8 aliphatics, 75% C9-C12 aliphatics and 0% C9-C16 aromatics adopted by HDOH for use in their guidance was selected. This generates a carbon range-weighted, TPH RfC for middle distillate vapors (TPHd) of 130 µg/m^3^:

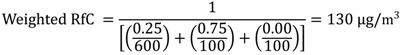
(7)


Note that the HDOH study did not identify a significant proportion of aliphatic compounds greater than C12 and aromatic compounds greater than C10 at any of the sites investigated. Laboratory-based studies have suggested a dominance of heavier compounds in vapors from some middle distillate fuels, however [26]. This would not significantly alter the weighted RfC for middle distillate vapors, since the toxicity of these compounds is assumed to be identical to medium-weight aliphatics and aromatics (see Table 4).

**Table 7:** Example, indoor air and soil vapor screening levels for TPH based on default, carbon range compositions for gasolines and middle distillates noted in text.

Fuel Type	Weighted RfC (µg/m^3^)	Indoor Air ^1^		Subslab Soil Vapor ^2^	
		Residential (µg/m^3^)	Commercial/Industrial (µg/m^3^)	Residential (µg/m^3^)	Commercial/Industrial (µg/m^3^)
Gasolines	279	290	410	290,000	810,000
Middle Distillates	130	140	190	140,000	380,000

^1^ Based on noncancer Hazard Quotient of 1.0; ^2^ Based on indoor air-soil vapor (subslab) attenuation factors of 0.001 for residential structures and 0.0005 for commercial/industrial structures (for example only) (after [8]).

The weighted, TPH toxicity factors for gasoline and diesel vapors can now be used to calculate TPHg and TPHd screening levels for indoor air and soil vapor in the same manner as done for individual compounds. Total Petroleum Hydrocarbon screening levels based on the equations and exposure assumptions discussed earlier are presented in Table 7. These screening levels can now be used to estimate “critical ratios” where the proportion of TPH in vapors in comparison to individual, targeted compounds such as benzene reaches a point that TPH will drive vapor intrusion risk.

### 2.6. Calculation of TPH Critical Ratios

The relative risk posed by two (or more) different chemicals under a given exposure pathway (e.g., vapor intrusion) is in part a function of toxicity and concentration. Aliphatic compounds that dominate TPH are, for example, significantly less toxic than benzene at equivalent exposure concentrations. This can be seen by a simple comparison of indoor air and soil vapor screening levels for carbon ranges and benzene in Table 6, Table 7. At some “critical ratio”, however, the overwhelming proportion of TPH in the vapors will override the risk posed by benzene and TPH will “drive” vapor intrusion risk. (Note that the term “risk” is used in a generic fashion to denote “noncancer hazard” and/or “excess cancer risk.”)

This ratio represents the weighted, indoor air, TPH screening level calculated for the samples divided by the indoor air screening level for benzene. If the ratio of TPH to benzene in soil vapor measured in the field exceeds this value, then the concentration of TPH in indoor air (or soil vapor) would in theory still exceed its risk-based screening level even though the concentration of benzene was at or below its respective screening level. If the critical ratio is not exceeded, then the concentration of TPH in indoor air (or soil vapor) would be at or below its respective screening level when the screening level for benzene is met. In the first case, TPH can be said to “drive” vapor intrusion risk, since screening and/or remediation of a site to address TPH vapors would coincidentally address potential vapor intrusion risks posed by benzene. In the second case, benzene can be said to drive vapor intrusion risk (*i.e.*, potential vapor intrusion risks posed by TPH would be adequately addressed at the point that the risk posed by benzene is addressed. This assumes, among other factors, that the average ratio of TPH to benzene calculated for the samples reflects the ratio in subslab soil vapor at the point that vapors intrude an overlying building.

As noted in Table 6, screening levels for TPH in indoor air or soil vapor can be up to 2,032 times higher than screening levels for benzene (e.g., C5-C8 aliphatic indoor air screening level of 630 µg/m^3^ divided by benzene indoor air screening level of 0.31 µg/m^3^ = 2,032). In this case, TPH will always drive vapor intrusion risk when the TPH:Benzene ratio exceeds 2,032:1, even if a conservative, target risk of 10^−^^6^ is applied to benzene. Similarly, screening levels for TPH can be almost 8,750 times higher than screening levels for naphthalene (*i.e.*, maximum TPH indoor air screening level of 630 µg/m^3^ divided by minimum naphthalene indoor air screening level of 0.072 µg/m^3^). This ratio will decrease as the proportion of longer-range aliphatics in petroleum vapors increases, along with the toxicity of the TPH vapors in general (*i.e.*, less TPH required to drive vapor intrusion risk over individual compounds). 

Table 8 presents a summary of critical ratios for TPH and individual compounds based on the example, indoor air and soil vapor screening levels presented in Table 6, Table 7 and the assumed, carbon range makeup of TPH vapors for gasoline and middle distillate fuels presented in Table 2. 

**Table 8:** Example critical ratios over which TPH in soil vapor will drive vapor intrusion risk over individual compound.

Chemical	Critical Ratio ^1,2^	
	TPH Gasoline Vapors	TPH Middle Distillate Vapors
Benzene	935	452
Ethylbenzene	299	144
Toluene	0.06	0.03
Xylenes	2.9	1.4
Naphthalene	4,028	1,944

^1^ TPH vapor intrusion screening level (Table 7) divided by individual compound screening level (Table 6); ^2^ Ratio at which TPH will exceed vapor intrusion screening level when individual compound is at or below its respective screening level (based on a target cancer risk of 10^−^^6^ or a noncancer Hazard Quotient of 1.0).

A critical ratio of 935:1 (290 µg/m^3^/0.31 µg/m^3^) is generated for TPH:Benzene, based on an assumed TPH vapor composition of 75% C5-C8 aliphatic compounds and 25% C9-C12 aliphatic plus aromatic compounds. The TPH critical ratios are reduced by a factor of two for vapors associated with diesel and other middle distillate fuels (*i.e.*, less TPH required to drive risk over individual compounds), based on an assumed TPH vapor composition of 25% C5-C8 aliphatic compounds and 75% C9-C12 aliphatic and C9-C10 aromatic compounds.

Default or site-specific critical ratios provide a very simple and quick tool to determine the potential significance of TPH as a vapor intrusion risk driver at a site where both TPH and benzene soil vapor data are available. For example, if the TPH:Benzene ratio exceeds 2,032:1 at a site then TPH will *always* drive vapor intrusion risk over benzene, regardless of the carbon range makeup of the TPH (*i.e.*, even if TPH is composed of 100% C5-C8 aliphatics) and even if a conservative, excess cancer risk of 10^−^^6^ is applied to benzene. The same is true when the TPH:Naphthalene ratio exceeds 8,750:1. In such cases, TPH vapors could still pose a vapor intrusion risk even though screening levels for individually targeted compounds are met. The lowest possible TPH:Benzene critical ratio using a benzene target risk of 10^−^^6^ is 323:1, based on a TPH vapor composition of 100% C9-C12+ aliphatics and/or C9-C10 aromatics (*i.e.*, 100 µg/m^3^ divided by benzene indoor air screening level of 0.31 µg/m^3^; see Table 6). In this example, TPH *could* drive vapor intrusion risk over benzene at a TPH:Benzene ratio as low as 323:1, depending on the actual carbon range makeup and weighted toxicity of the TPH. 

Similar, example critical ratios were calculated for other targeted compounds (*i.e.*, TEXN). The ratio increases for compounds that are more toxic than benzene (e.g., naphthalene critical ratio 8,750:1) and decreases for compounds that are less toxic (e.g., toluene critical ratio 0.06:1). In other words, a higher proportion of TPH in soil vapor (or indoor air) is required to overwhelm the vapor intrusion risk posed by an individual compound as the toxicity of the targeted compound increases.

The relative role of TPH in vapor intrusion risk will ultimately depend on the actual carbon range chemistry of the TPH and the associated toxicity and the target risk used to screen for individual compounds. Less TPH is required to overwhelm the risk posed by an individual chemical as the proportion of more toxic, C9-C18 aliphatics (or C9-C16 aromatics) increases. Critical ratios are also necessarily dependent on the toxicity factors applied to individual, TPH carbon ranges. Toxicity factors published by the State of Massachusetts [19], for example, are more conservative than USEPA toxicity factors by a factor of two to three [16]. Critical ratios based on Massachusetts toxicity factors would be lower (*i.e.*, more conservative) by a similar amount.

In the next section of this paper, these screening tools are applied to the soil vapor database compiled by the USEPA and to a separate petroleum vapor study carried out by the State of Hawaii in order to evaluate the relative role of TPH in vapor intrusion at petroleum-contaminated sites. The first database focuses on soil vapor sample data from purported gasoline releases. The Hawaii study focuses primarily on soil vapor data from middle distillate releases, and serves as a supplement to the USEPA database.

## 3. Application of Method to Case Studies

### 3.1. Selection of Representative Case Studies

In the previous sections we reviewed the basic chemistry and toxicity of petroleum vapors in terms of TPH carbon ranges and targeted, individual compounds such as benzene. We presented published toxicity factors for carbon ranges and summarized the approach for calculation of risk-based, indoor air and soil vapor screening levels, including screening levels for TPH in general. We then presented the concept of “critical ratios” of TPH to individual, targeted compounds that can be used to quickly assess the relative role of TPH in potential vapor intrusion threats on a site-by-site basis. 

In the following discussions, we apply these tools to two sets of case studies for petroleum-contaminated sites in order to answer the ultimate question posed at the beginning of this paper: “Do field data support conditions where vapor intrusion concerns posed by petroleum could be driven by the TPH rather by individual compounds such as benzene?” Data are first screened in terms of TPH:Benzene ratios and the potential for TPH to play a significant role in vapor intrusion risk reviewed. The carbon range makeup of the TPH is then evaluated in more detail. Weighted, TPH reference doses are then used to calculate more site specific (or database-specific), TPH screening levels for indoor air and soil vapor and the data re-evaluated. 

The first set of case studies reflect a soil vapor sample data set being compiled by the USEPA for primarily gasoline-contaminated sites. The second set of case studies and data are based on a study carried out by the State of Hawaii under a grant from the USEPA for sites contaminated with diesel and other middle distillate fuels. The sites included in the Hawaii study were targeted to fill in gaps in the USEPA database and more closely evaluate the potential for non gasoline-contaminated sites to pose potential vapor intrusion threats.

Both data sets focus primarily on the nature of petroleum vapors within the immediate vicinity of the source area (*i.e.*, within fifteen feet of contaminated soil or groundwater). The fate and transport of vapors at increasing distances from the source areas is not directly reviewed, although characteristics such as the ratio of TPH to key, indicator compounds such as benzene can shed light on this subject.

The reviews presented below are intended for illustration purposes only and are not intended to be a comprehensive evaluation of the sites involved. The USEPA data are, for example, summarized in terms of individual sample points rather than the range and average for sites. This introduces a potential bias toward sites with a higher number of sample points in comparison to those with only a few sample points. For the purposes of this paper it is assumed that this bias is small and that the data in general are adequately representative.

### 3.2. Vapors Associated with Gasolines

As introduced earlier, the USEPA Office of Underground Storage Tanks (UST) has compiled a “Petroleum Vapor Intrusion” database of soil vapor data for seventy sites in the US, Canada and Australia [32]. The database focuses on known or presumed, gasoline-contaminated sites associated with releases from USTs. Although limited in terms of the total number of petroleum release sites in these countries, in the hundreds of thousands in the US alone, the database provides a useful snapshot of the chemistry of vapors associated with gasoline-contaminated sites. A summary of data used in the following evaluation of the database is provided in the supplement to this paper. 

**Figure 2:**
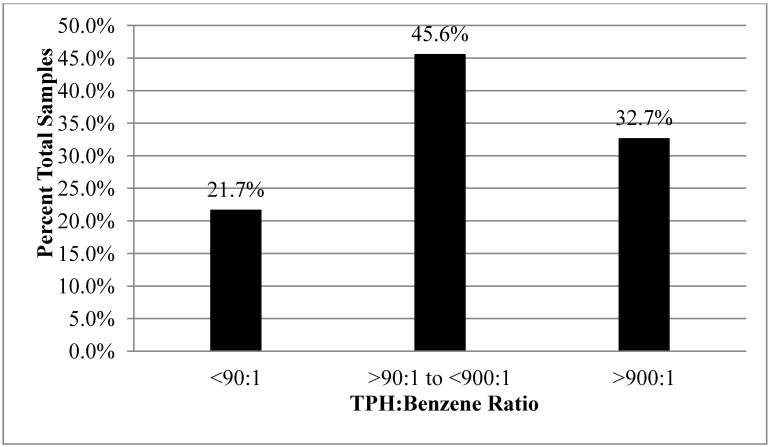
Summary of TPH to benzene ratios for soil vapor samples included in the USEPA PVI database (n = 364). Reflects gasoline-only sites with >1,000 µg/m^3^ TPH.

Figure 2 presents a summary of TPH-to-Benzene ratios for soil vapor samples included in the USEPA PVI database. As discussed earlier, only samples with reported concentrations of TPH greater than 1,000 µgm^3^ were considered in order to limit potential biases due to laboratory detection limits or interference from outdoor air [2]. A total of 364 samples met these criteria and included data for both TPH and benzene (see paper supplement). The inclusion of benzene in reported TPH concentrations is not known. The consistently high ratio of TPH to benzene in the samples negates a significant bias with respect to double counting of benzene in the TPH data. Non-specific, TPH hydrocarbon compounds clearly dominate petroleum vapors in the samples included in the USEPA database. The ratio of TPH to benzene ratio is consistently greater than 4:1, however, with a median ratio of 301:1, an average of 5,566:1 and a high of 4,000,000:1. The TPH:Benzene ratio varies by an order of magnitude or more at most sites where multiple samples were collected and up to three orders of magnitude at some sites (see supplement). The potential causes of this variability are discussed below.

As depicted in Figure 2, the ratio of TPH to benzene exceeds the default, critical ratio of 900:1 (rounded from 935:1, see Table 8) developed earlier for gasoline vapors in 33% of the samples included the database. This implies that the overwhelming proportion of aliphatic compounds in these samples would cause TPH, and not benzene, to drive potential vapor intrusion risks. In other words, if vapor intrusion were indeed a concern at these sites (e.g., subslab soil vapor screening levels exceeded and intrusion pathways present), then remediation of the site to reduce benzene in soil vapor down to target screening levels may not adequately address the noncancer risk posed by the TPH component of the vapors. Screening and/or remediation of the site to address TPH concerns would, however, concurrently address vapor intrusion concerns associated with benzene (*i.e.*, benzene would be below respective screening level at the point that TPH screening level was met).

Recall that this ratio assumes a target risk for benzene of 10^−^^6^ and a correlatively conservative indoor air and subsequent soil vapor screening level (e.g., target indoor air goal of 0.31 µg/m^3^ for residential scenarios; see Table 6). If a less conservative, target risk were used to calculate screening levels then the risk of missing potential vapor intrusion problems posed by TPH would be much higher. For example, the critical TPH:Benzene ratio associated with a target risk of 10^−^^5^ for the latter would be 90:1, adjusting the previous example downward by a factor of ten (*i.e.*, 290 µg/m^3^ divided by 3.1 µg/m^3^). In the case of the samples referenced from the USEPA database, the TPH:Benzene ratio exceeds this critical ratio 78% of the time (see Figure 2). This highlights the importance of quantitatively including TPH in vapor intrusion studies when a less conservative, target risk and associated screening levels are applied for individual compounds such as benzene. Note that this is not affected by attenuation factors assumed in the screening levels, since they are presumably identical for both benzene and TPH.

The relatively high proportion of TPH to benzene for a significant number of vapor samples from gasoline-only sites included in the USEPA database was initially surprising, given the traditional focus on only the BTEX fraction of these fuels [33]. As discussed earlier, seemingly low levels of benzene in the samples could be due to a number of factors, including: (1) Inadvertent inclusion of vapor data associated with middle distillate fuels in the database, (2) An original, minimal concentration of benzene in the gasoline released, (3) Preferential removal of benzene from soil vapors due to partitioning into soil moisture, and/or preferential biodegradation. Given the relatively high concentration of TPH reported in the samples (up to 31,000,000 µg/m^3^), the dominance of C5-C8 aliphatics over C9-C12 aliphatics in seven of nine samples with carbon range data and a TPH:Benzene ratio >900:1 (see supplement), and laboratory studies that suggest a much lower biodegradation rate for aromatics than aliphatics [34], the most likely cause for at least some of the samples appears to be an initially low concentrations of benzene in the gasoline released at the site. Likely variation in the degradation and removal of aliphatic and aromatic compounds between and even within sites complicates interpretation of the data. A more detailed study of this issue is beyond the scope of this paper.

As discussed earlier, several oil companies have moved toward low-benzene gasolines in recent years in order to lower the toxicity of auto exhaust as well as soil and groundwater contaminated by inadvertent releases of the fuels. Releases associated with some of these fuels appear to have been captured in the USEPA database. This is an important observation, given a common assumption that benzene can be used as a stand-alone tool to evaluate the risk posed by releases of gasoline to the environment, including vapor intrusion (e.g., see [32]). This evaluation appears to have focused on traditionally targeted, individual compounds and did not specifically consider the relative role of TPH in vapor intrusion. Indeed, the TPH:Benzene ratio exceeds the maximum critical ratio of 2,032:1 in 24% of the soil vapor samples from supposed gasoline-only sites (see supplement). This implies that TPH would drive vapor intrusion risk over benzene regardless of both the target risk applied to benzene (e.g., 10^−^^6^ excess cancer risk) *and* the carbon range composition of the TPH vapors (e.g., best case 100% C5-C8 aliphatics).

### 3.3. Vapors Associated with Diesel and Other Middle Distillate Fuels

The PVI database being compiled by the USEPA focuses on vapors associated with gasoline-contaminated soil and groundwater. As presented earlier, the Hawaii Department of Health (HDOH), through a grant from the USEPA, carried out a field study of the chemistry and toxicity of vapors associated soil and groundwater contaminated with diesel and other middle distillate fuels in an effort to supplement the USEPA database [10]. Particular emphasis was placed on the aliphatic and aromatic makeup of the TPH component of petroleum vapors and the potential for TPH to drive potential vapor intrusion risk over individual compounds such as benzene, toluene, ethylbenzene, xylenes and naphthalene.

Soil vapor data for petroleum-contaminated sites across Hawaii were reviewed as part of the study. Five sites with known, heavy contamination were targeted for detailed sampling. A limited number of samples were also collected over fresh fuels, although these data are not reviewed as part of this paper. Fuels released at sites included gasolines, including AVGAS and JP-4, JP-8 and diesel. Pipeline releases with widespread contamination and existing soil vapor monitoring points were targeted in order to ensure that vapors would be encountered and to minimize field sample collection costs. Sites A, B, C and E are believed to reflect a progressive domination by diesel and/or other middle distillate fuels such as JP-8 (similar to diesel). Site D is associated with a forty year-old release of JP-4 (mix of gasoline and kerosene) from a large fuel pipeline.

TPH compounds dominated petroleum vapors at each of the five, primary sites investigated during the study as well as other sites reviewed during the study, with less than 1% of the total vapors generally attributable to BTEXN (Table 9). The average ratio of TPH to benzene in soil vapors ranged from 1,500:1 at a site contaminated with JP-4 and AVGAS to over 18,000:1 at a site contaminated primarily by diesel fuel. The average TPH:Benzene ratio exceeded 2,000:1 at the three sites where diesel and other middle distillate fuels were known to be present. As noted in Table 9, the maximum concentration of TPH in soil vapor samples collected at the sites were well above screening levels ultimately generated for potential vapor intrusion concerns.

The overwhelming proportion of TPH in the soil vapors at these sites ensure that TPH will dominate vapor intrusion risks over benzene and other individual VOCs regardless of the actual carbon range makeup and weighted toxicity of the TPH, even if a conservative, target risk were used for carcinogens. The average TPH:Benzene ratio at an aged, JP-4/AVGAS release site included in the study ( >9,000:1; Site A) exceeded the default, critical ratio for gasoline vapors of 900:1 noted earlier. The TPH:Benzene ratio for soil vapor samples collected at middle distillate sites was even higher. The near absence of benzene in soil vapors at the JP-4/AVGAS site could be associated with a preferential removal of vapor-phase, aromatic compounds over aliphatic compounds over time due, for example, to preferential diffusion into soil moisture. This could also be simply due to an absence of significant benzene in the original fuels released. Similar observations have been made at other gasoline-contaminated sites in Hawaii [10].

**Table 9:** Example TPH concentration in soil vapor, average TPH:Benzene ratio and TPH carbon range makeup of soil vapor samples collected in the Hawaii DOH petroleum vapor study (based on summa canister, TO-15 data).

Site/Fuel Type	ExampleTPH(μg/m^3^)	AverageTPH:Benzene Ratio	Average Carbon Range Composition		
			Aliphatics		Aromatics
			C5-8	C9-10	C9-12
Site A (JP-4/AVGAS)	300,000,000 μg/m^3^	1,513:1	96%	0.2%	3.3%
Site B (mixed fuels)	220,000,000 μg/m^3^	4,174:1	93%	0.3%	6.8%
Site C (JP-8 +/− JP-4)	86,000,000 μg/m^3^	18,710:1	72%	0.6%	27%
Site D (JP-4/AVGAS)	2,600,000 μg/m^3^	9,135:1	63%	4.1%	33%
Site E (diesel)	13,000,000 μg/m^3^	54,236:1	25%	0.9%	74%

Aliphatic compounds dominate TPH vapors at all of the sites, although the relative proportion of C5-C8 *versus* C9-C12 compounds varied considerably (see Table 9). A comparison of co-located and concurrent Summa canister data to sorbent tube data identified only a minor contribution of C13+ aliphatic compounds for TPH vapors at the sites (<10%). The contribution of C9 and higher, aromatic TPH compounds in the samples was likewise negligible.

Weighted TPH Reference Concentrations and associated indoor air and soil as screening levels based on the carbon range makeup of the TPH follow a similar trend (Table 10). The weighted TPH RfC and associated action levels calculated for vapors associated with a relatively recent, gasoline-contaminated site (e.g., Site A and Site B) approach those for C5-C8 aliphatics (e.g., TPH RfC 400 to 600 µg/m^3^). The weighted TPH RfC and associated action levels calculated for vapors collected from sites progressively dominated by diesel or other middle distillate fuels (Sites B, C and E) or associated with aged, JP-4 (Site D) approach those for the more toxic, C9-C12 aliphatic compounds (e.g., TPH RfC 100 to 200 µg/m^3^) and are reflective of the higher proportion of these compounds in the vapors.

The lowest (*i.e.*, most “toxic”), weighted Reference Concentration calculated was calculated for samples collected from an aged, diesel-contaminated site where TPH vapors were composed of an average 75% C9-12 aliphatics (Site E in Table 10). Free product on groundwater at the site was relatively shallow (<10 ft). Concentrations of TPH in soil vapor were perhaps an order of magnitude lower than would be anticipated at a site contaminated to a similar amount of gasoline. Even so, TPH in some samples exceeded 100,000,000 μg/m^3^, and were well above screening levels for potential vapor intrusion concerns.

**Table 10:** Weighted TPH Reference Concentration and example TPH subslab soil vapor screening levels for soil vapor samples collected in the Hawaii DOH petroleum vapor study.

Site/Fuel Type	Weighted RfC ^1^(μg/m^3^)	Indoor AirScreening Level ^2^(μg/m^3^)	Subslab Soil VaporScreening Level ^3^(µg/m^3^)	TPH:Benzene Critical Ratio ^4^	TPH:Benzene Measured Ratio	Vapor Intrusion Risk Driver ^5^
Site A	510	530	530,000	1,710:1	1,513:1	Benzene
Site B	443	460	460,000	1,484:1	4,174:1	TPH
Site C	251	260	260,000	839:1	18,710:1	TPH
Site D	211	220	220,000	710:1	9,135:1	TPH
Site E	127	130	130,000	410	54,236:1	TPH

^1^ Based on average carbon range composition (see Table 9); ^2^ Residential exposure scenario; see equation and assumptions in text; ^3^ Assuming an indoor air:subslab soil vapor attenuation factor of 0.001; ^4^ TPH indoor air screening level divided by benzene screening level (based on target cancer risk of 10^−^^6^); Above this ratio, TPH in soil vapor could still pose a vapor intrusion risk even if benzene is at or below target screening levels. ^5^ Based on comparison to average TPH: Benzene ratio for samples noted in previous table.

The TPH:Benzene critical ratio for each set of study site samples is noted in Table 10. A comparison of these ratios to the measured, TPH:Benzene ratio for samples collected at each site provides insight on the relative role of TPH in overall vapor intrusion risk. As indicated in Table 10, benzene drives potential vapor intrusion risk over TPH for soil vapor samples collected at Site A, a JP-4/AVGAS release (*i.e.*, measured TPH:Benzene ratio in soil vapor below critical ratio). Dividing the measured TPH:Benzene ratio by the risk-based, critical ratio for the same samples represents the theoretical, noncancer Hazard Quotient for TPH with respect to vapor intrusion at the point that the concentration of benzene in soil vapor equals the target, benzene screening level. In the case of Site A, a Hazard Quotient of 0.9 is calculated, suggesting that TPH will not pose a significant vapor intrusion risk if a target, 10^−^^6^ risk is met for benzene. Note that use of a target risk of 10^−^^5^ to screen for benzene would be associated with a theoretical, noncancer Hazard Quotient of approximately nine for TPH. This highlights the need to use a conservative, target cancer risk for benzene at sites with the measured, TPH:Benzene ratio of more than approximately 100:1, as a rough guide.

It is interesting to note that screening and/or remediation of Site A with respect to TPH only and without consideration of benzene would at worst leave benzene in soil vapors only marginally above the target, 10^−^^6^ risk goal. Reducing TPH in soil vapor to 530,000 µg/m^3^ would in theory result in a concentration of benzene in soil vapor of approximately 350 µg/m^3^, only marginally above the screening level of 310 µg/m^3^ and equating to a cancer risk of only 1.1 × 10^−^^6^. Ignoring benzene and focusing only on TPH would be unlikely to leave potentially significant, vapor intrusion risks posed by the former unaddressed.

A comparison of the TPH to benzene field ratio to the calculated, risk-based, critical ratio at the remaining four sites included in the Hawaii study clearly identifies TPH as the vapor intrusion risk driver. For samples collected from Site B, the measured TPH:Benzene ratio exceeds the risk-based, critical ratio for the same sample set by a factor of almost three (see Table 9, Table 10). In theory, this suggests that the noncancer, Hazard Quotient posed by TPH in soil vapor for vapor intrusion would still approach three at the point that the concentration of benzene was reduced to a target, 10^−^^6^ risk (*i.e.*, TPH in soil vapor would equal approximately 1,300,000 µg/m^3^ at the point that benzene equals 310 µg/m^3^). The TPH:Benzene critical ratio is exceeded by an even larger degree for samples collected at the remaining three sites (*i.e.*, twenty-two, thirteen and one-hundred thirty two for Sites C, D and E, respectively). This suggests that TPH could still pose a significant vapor intrusion hazard at the sites well beyond the point that a target risk of 10^−^^6^ for benzene was met. This is not surprising, given the relatively minor contribution of benzene to overall petroleum vapors at the sites. It is also worthwhile to note that naphthalene and methylnaphthalenes played a limited role in potential vapor intrusion risk at the middle distillate sites reviewed in the study, in spite of the assumed higher concentration of these chemicals in the original fuel released. The lack of significant naphthalenes in soil vapor samples is most likely due to the propensity of these chemicals to sorb to soil particles rather than partition into the vapor phase.

The Hawaii study highlights the potential for significant, vapor intrusion concerns posed by subsurface releases of middle distillate fuels, including diesel, as well as low-benzene gasolines. Reported concentrations of TPH in shallow soil vapor samples collected within or near source areas were well above risk-based screening levels for vapor intrusion concerns. The study also highlights the need to quantitatively consider TPH in vapor intrusion risk assessments at these sites when the ratio of TPH to benzene in soil vapor exceeds a value of approximately 450:1 if a target risk of 10^−^^6^ is applied to benzene or a value of approximately 45:1 if a target risk of 10^−^^5^ is applied (e.g., TPH indoor air screening level of 140 µg/m^3^ divided by benzene screening level of 0.31 µg/m^3^ or 3.1 µg/m^3^; see Table 6, Table 7).

## 4. Summary and Conclusions

Vapors emitted from petroleum fuels are dominated by aliphatic and to a lesser degree aromatic compounds collectively measured as Total Petroleum Hydrocarbons or “TPH”. Published physiochemical constants and toxicity factors for volatile, TPH aliphatic and aromatic carbon ranges allows for quantitative, risk-based evaluation of TPH in vapor intrusion investigations in the same manner as carried out for traditionally targeted chemicals such as benzene, toluene, ethylbenzene, xylenes and naphthalene. Generic and/or site-specific TPH screening levels can be generated based on the assumed or known aliphatic and aromatic makeup of the petroleum vapors.

The relative role of TPH in vapor intrusion in comparison to individually targeted compounds such as benzene can be quickly determined by comparison of the ratio of TPH to the compound measured in the field to the ratio of risk-based screening levels for these chemicals. If, for example, the ratio of TPH to benzene in soil vapor measured in the field exceeds this “critical ratio” based on a comparison of screening levels then the concentration of TPH in indoor air (or soil vapor) would still exceed its risk-based screening level even though the concentration of benzene was at or below its respective screening level. If the critical ratio is not exceeded, then the concentration of TPH in indoor air (or soil vapor) would be at or below its respective screening level when the screening level for benzene is met. In the first case, reliance on benzene data alone to assess potential vapor intrusion risks would be inappropriate. In the latter case, a focus on benzene for final decision making purposes should ensure that potential vapor intrusion risks posed by TPH will also be addressed. 

Critical ratios are necessarily dependent on the toxicity factors applied to individual, TPH carbon ranges. Based on TPH toxicity factors published by the USEPA [16] and a 10^−^^6^ excess cancer risk for benzene, a TPH:Benzene critical ratio of approximately 900:1 serves as a conservative tool for initial screening of gasoline-contaminated sites (*i.e.*, TPH could drive vapor intrusion risk when the concentration of TPH is more than 900 times that of benzene). This ratio is not exceeded for the majority (67%) of samples from gasoline-contaminated sites included a soil vapor database compiled by the USEPA [32]. This suggests that consideration of benzene in the absence of TPH data will be adequate to screen most gasoline-contaminated sites for potential vapor intrusion concerns if a conservative target cancer risk is applied to benzene. 

Benzene clearly drives vapor intrusion risk for only 22% of the samples in the USEPA database, however, if a less conservative target risk of 10^−^^5^ is applied (*i.e.*, order-of-magnitude higher concentration of benzene considered acceptable). Furthermore, the measured ratio of TPH to benzene exceeded the screening value of 900:1 for 33% of the samples in the database, implying that TPH could drive vapor intrusion risk over benzene with respect to these samples depending on the target risk applied to the latter and the actual carbon range makeup of TPH. At least some of these sites appear to be associated with releases of gasoline that was originally low in benzene. In addition, the TPH:Benzene ratio exceeds a hypothetical, toxicity-based, maximum critical ratio of 2,032:1 in 24% of the soil vapor samples in the USEPA database. This implies that TPH would drive vapor intrusion risk over benzene regardless of both the target risk applied to benzene *and* the carbon range composition of the TPH vapors. 

Initial screening of gasoline-contaminated sites with respect to relative proportions of TPH and benzene present in soil vapors therefore appears to be prudent. Note that this may appear to conflict with the statement in the USEPA PVI database report that “available data indicate benzene is the risk driver for the (gasoline-release) sites evaluated” [32]. This conclusion however, was based on a comparison of the relative vapor intrusion risk posed by benzene to other, traditionally targeted, individual compounds such as toluene, ethylbenzene, xylenes and naphthalene. A detailed evaluation of the TPH component of the PVI database had not been carried out at the time that the USEPA report was published. This paper expands the database evaluation to include this comparison.

Vapors associated with subsurface releases of diesel and other middle distillate fuels can exhibit a higher proportion of more toxic, C9-C12 and higher aliphatic compounds, although the magnitude of vapors released from contaminated soil and groundwater will be lower than for an equivalent amount of gasoline. In this case a lesser amount of TPH in soil vapor (or indoor air) is required before the TPH fraction of the vapors begins to drive vapor intrusion risk over benzene or other individual compounds. Based on a limited study carried out by the State of Hawaii, a critical TPH to benzene ratio of approximately 450:1 served as a useful tool for initial screening of vapor data at sites contaminated with diesel or other middle distillate fuels. The measured ratio of TPH to benzene at all of the middle distillate sites reviewed in the Hawaii study reviewed in this paper exceeded this ratio by a wide margin, suggesting that TPH will play a dominant role in vapor intrusion at sites contaminated by these types of fuels. Significant levels of both C5-C8 aliphatics and C9-C12 aliphatics at the sites investigated highlight the need to report TPH as the sum of C5-C12 compounds for soil vapor samples collected at middle distillate-release sites, even though this is traditionally referred to as “gasoline range” hydrocarbons by commercial laboratories.

Carbon range data for TPH in soil vapor can be used to develop site-specific vapor intrusion screening levels for TPH or for direct calculation of potential vapor intrusion risk. A review of case studies highlights the importance of including a review of TPH in vapor intrusion investigations. This can be done at an initial screening level by simple comparison of the measured ratio of TPH to benzene and other targeted compounds to the ratio of generic or site-specific, risk-based screening levels for these compounds. The gradual reduction of benzene in gasolines over time and high concentrations of aliphatic compounds in vapors associated with diesel releases highlights the need to consider TPH in vapor intrusion studies.

Identification of TPH or individual compounds in soil vapor above target screening levels and/or critical ratios does not necessarily imply that a vapor intrusion problem indeed exists. It is worthwhile to note that odor thresholds for petroleum fuels are within an order of magnitude of the risk-based screening levels for TPH presented in this paper. Given the hundreds of thousands of petroleum releases identified in the US over the past twenty years, the fact that few instances of petroleum-related vapor intrusion have been reported suggests in itself that significant risks are most likely limited to the presence of heavy contamination in soil or groundwater within close proximity to a building floor. 

As discussed in numerous studies, this suggests that significant attenuation forces beyond those typically assumed for chlorinated solvents are in play both beneath and most likely within the subject buildings. Natural biodegradation of vapor-phase, petroleum compounds in contaminated soil and groundwater will significantly reduce the long-term vapor-intrusion risk of subsurface contamination in comparison to soil contaminated with an equal amount of chlorinated solvents. Regional climate, geology and associated building ventilation designs strongly influence local indoor air: subslab attenuation factors. The relative persistence of petroleum compounds in indoor air with respect to vapor flux rates should also be considered.

## Supplementary Files

Supplementary File 1 Supplementary (XLSX, 81 KB)
