# *Correction*: Brewer, R.; *et al.* Risk-Based Evaluation of Total Petroleum Hydrocarbons in Vapor Intrusion Studies. *Int. J. Environ. Res. Public Health* 2013, *10*, 2441–2467

**DOI:** 10.3390/ijerph110707182

**Published:** 2014-07-14

**Authors:** Roger Brewer, Josh Nagashima, Michael Kelley, Marvin Heskett, Mark Rigby

**Affiliations:** 1 Hawaii Department of Health, 919 Ala Moana Blvd Room 206, Honolulu, HI 96814, USA; E-Mail: josh.nagashima@doh.hawaii.gov; 2 EA Engineering, Science and Technology, 615 Piikoi St #515, Honolulu, HI 96814, USA; E-Mail: mkelley@eaest.com; 3 Geotek Hawaii, Inc., P.O. Box 1555, Pearl City, HI 96782, USA; E-Mail: mhesketts@mac.com; 4 Parsons Corporation, 10235 South Jordan Gateway, Suite 300, South Jordan, UT 8409, USA; E-Mail: mark.rigby@parsons.com

The authors wish to add the following amendments and corrections to their paper published in *IJERPH* [1].

Page 2460, Table 9: The data in last two columns are reversed. The correct Table 9 should be:

**Table 9:** Example TPH concentration in soil vapor, average TPH:Benzene ratio and TPH carbon range makeup of soil vapor samples collected in the Hawaii DOH petroleum vapor study (based on summa canister, TO-15 data).

Site/Fuel Type	Example TPH (μg/m^3^)	Average TPH:Benzene Ratio	Average Carbon Range Composition		
			Aliphatics		Aromatics
			C5-8	C9-12	C9-10
Site A (JP-4/AVGAS)	300,000,000 μg/m^3^	1,513:1	96%	3.3%	0.2%
Site B (mixed fuels)	220,000,000 μg/m^3^	4,174:1	93%	6.8%	0.3%
Site C (JP-8 +/− JP-4)	86,000,000 μg/m^3^	18,710:1	72%	27%	0.6%
Site D (JP-4/AVGAS)	2,600,000 μg/m^3^	9,135:1	63%	33%	4.1%
Site E (diesel)	13,000,000 μg/m^3^	54,236:1	25%	74%	0.9%

The authors would like to apologize for any inconvenience caused to the readers by these changes.

## References

[1] Brewer R., Nagashima J., Kelley M., Rigby M. (2013). Risk-based evaluation of total petroleum hydrocarbons in vapor intrusion studies. Int. J. Environ. Res. Public Health.

